# Molecular Pathology Demonstration of SARS-CoV-2 in Cytotrophoblast from Placental Tissue with Chronic Histiocytic Intervillositis, Trophoblast Necrosis and COVID-19

**DOI:** 10.3390/jdb9030033

**Published:** 2021-08-25

**Authors:** David A. Schwartz, Mattia Bugatti, Amerigo Santoro, Fabio Facchetti

**Affiliations:** 1 Department of Pathology, Medical College of Georgia, Augusta University, Augusta, GA 30912, USA; 2 Pathology Unit, Department of Molecular and Translational Medicine, University of Brescia, 25123 Brescia, Italy; mattia.bugatti@unibs.it (M.B.); amerigo.santoro@asst-spedalicivili.it (A.S.); fabio.facchetti@unibs.it (F.F.)

**Keywords:** SARS-CoV-2, placental pathology, cytotrophoblast, syncytiotrophoblast, COVID-19, pregnancy, coronavirus placental infection, molecular pathology, chronic histiocytic intervillositis, SARS-CoV-2 in cytotrophoblast

## Abstract

A subset of placentas from pregnant women having the SARS-CoV-2 infection have been found to be infected with the coronavirus using molecular pathology methods including immunohistochemistry and RNA in situ hybridization. These infected placentas can demonstrate several unusual findings which occur together—chronic histiocytic intervillositis, trophoblast necrosis and positive staining of the syncytiotrophoblast for SARS-CoV-2. They frequently also have increased fibrin deposition, which can be massive in some cases. Syncytiotrophoblast is the most frequent fetal-derived cell type to be positive for SARS-CoV-2. It has recently been shown that in a small number of infected placentas, villous stromal macrophages, termed Hofbauer cells, and villous capillary endothelial cells can also stain positive for SARS-CoV-2. This report describes a placenta from a pregnant woman with SARS-CoV-2 that had chronic histiocytic intervillositis, trophoblast necrosis, increased fibrin deposition and positive staining of the syncytiotrophoblast for SARS-CoV-2. In addition, molecular pathology testing including RNAscope and immunohistochemistry for SARS-CoV-2 and double-staining immunohistochemistry using antibodies to E-cadherin and GATA3 revealed that cytotrophoblast cells stained intensely for SARS-CoV-2. All of the cytotrophoblast cells that demonstrated positive staining for SARS-CoV-2 were in direct physical contact with overlying syncytiotrophoblast that also stained positive for the virus. The pattern of cytotrophoblast staining for SARS-CoV-2 was patchy, and there were chorionic villi having diffuse positive staining of the syncytiotrophoblast for SARS-CoV-2, but without staining of cytotrophoblast. This first detailed description of cytotrophoblast involvement by SARS-CoV-2 adds another fetal cell type from infected placentas that demonstrate viral staining.

## 1. Introduction

The coronavirus disease 2019 (COVID-19) pandemic has continued to have a potential adverse effect on pregnant women and their infants. When the etiological agent, the novel coronavirus severe acute respiratory syndrome coronavirus 2 (SARS-CoV-2), was initially identified from pregnant women from China, it was hoped that vertical transmission of the virus would not occur, similar to previous coronavirus infections severe acute respiratory syndrome coronavirus (SARS-CoV) and Middle East respiratory syndrome coronavirus (MERS-CoV), and other RNA respiratory viruses [1,2]. Early reports of pregnancy outcomes from China were encouraging as no confirmed cases of vertical transmission were identified [3,4,5,6]. Concern remained for the possibility of vertical transmission, however, including whether SARS-CoV-2 could undergo maternal-infant transmission either by intrauterine or postpartum mechanisms [7,8,9,10,11]. With the spread of the virus throughout the globe and additional cases of SARS-CoV-2 infection in pregnancy being reported, it became evident that a small percentage of neonates delivered to infected mothers tested positive for SARS-CoV-2 [12,13,14,15]. This raised the questions of how and when these infants had acquired their infection, whether it was vertically transmitted, and if SARS-CoV-2 could be transmitted transplacentally prior to delivery from an infected pregnant woman to her fetus [16,17,18]. Criteria for the recognition of intrauterine transplacental infection from SARS-CoV-2 were proposed by Schwartz et al. [19], and a subset of cases were eventually identified as representing transmission of the virus across the placenta from an infected mother to the fetus [20,21,22,23,24]. By utilizing such molecular pathology methods as immunohistochemistry and RNA in situ hybridization [25], Schwartz and colleagues found that these transmitting placentas were infected with SARS-CoV-2 and shared a common and unique pattern of coexisting placental pathology abnormalities [26,27].

Placentas infected with SARS-CoV-2 have been found to have a group of three unusual pathology findings that occur together. These include chronic histiocytic intervillositis, trophoblast necrosis, and using molecular pathology methods, positivity of the syncytiotrophoblast for SARS-CoV-2 [26,27]. Among all placental cell types, the syncytiotrophoblast appears to be the most frequently involved cell type that stains for SARS-CoV-2 antigens or RNA [28]. To a much lesser extent, recent research has identified SARS-CoV-2 staining in placental stromal macrophages, termed Hofbauer cells [29], and endothelial cells of the villous capillaries in a small number of infected placentas [29]. In this report we provide immunohistochemical and molecular pathology evidence of SARS-CoV-2 staining in villous cytotrophoblast cells of the placenta which occurred in a placenta from a fetus having acquired the infection following transplacental transmission.

## 2. Materials and Methods

### 2.1. Clinical History 

A 31-year-old pregnant woman developed severe interstitial pneumonia associated with disseminated intravascular coagulation because of a COVID-19 infection and required hospitalization at 31 weeks 3 days gestational age. She had multiple positive tests for SARS-CoV-2 using reverse transcription polymerase chain reaction (RT-PCR). A cesarean section was performed that resulted in a female infant with a birthweight of 1615 g and an Apgar score of 1 at 1 min. She was intubated at 5 min, with slow recovery of cardio-respiratory function. Testing of the preterm neonate for SARS-CoV-2 using RT-PCR from nasal swabs was negative at 0 and 48 h after delivery. The placenta was sent for pathological evaluation. Both the mother and baby were discharged from the hospital 24 days following delivery in good condition.

### 2.2. Placental Pathology

The placenta was fixed in 10% buffered formalin and weighed 375 g following removal of the cord and membranes. The placenta appeared unremarkable upon gross examination, and after complete fixation representative sections of placenta were sampled according to the Amsterdam guidelines [30]. Following routine tissue histological processing, sections were stained with hematoxylin & eosin for microscopic diagnosis. 

### 2.3. Immunohistochemistry and Molecular Pathology 

Evaluation of the placenta was performed using several immunohistochemical techniques to evaluate for viral and host cell antigens as well as molecular pathology for SARS-CoV-2 nucleic acid using RNA in situ hybridization (RNAscope). The presence of histiocytes in the intervillous space and Hofbauer cells in the chorionic villous stroma was evaluated using antibody to CD68 and CD163 (Diagnostic BioSystems, Pleasanton, CA, USA). Identification of SARS-CoV-2 nucleocapsid antigen was evaluated by immunohistochemistry using monoclonal antibody to SARS-CoV-2 nucleocapsid protein (Sino Biological, Beijing, China) according to previously described methods [20]. To identify cytotrophoblast, two different antibodies were used: monoclonal mouse anti-human GATA3 antibody (clone L50-823, Cell Marque, Rocklin, CA, USA) and mouse monoclonal antibody to E-cadherin (Invitrogen clone 4A2C7, Thermo Fisher Scientific, Waltham, MA, USA). Double-staining immunohistochemistry was used to detect SARS-CoV-2 staining of the cytotrophoblast in which slides were stained with either GATA3 antibody or antibody to E-cadherin and antibody to SARS-CoV-2 nucleocapsid protein. 

RNA in situ hybridization for detection of SARS-CoV-2 spike protein mRNA (Advanced Cell Diagnostic V-nCov2019-S-antisense probe, Newark, CA, USA) and the RNAscope 2.5 HD Detection Reagent-Red (Advanced Cell Diagnostic) was performed using methods that have been previously described [20]. 

## 3. Results

**Figure 1:**
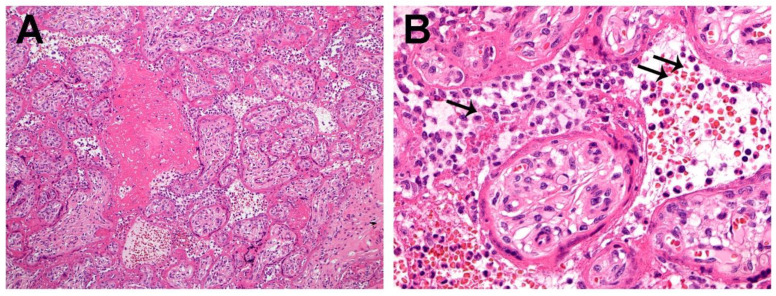
(**A**). This low magnification image of the placenta demonstrates diffuse trophoblast necrosis. The intervillous space has extensive fibrin deposition and chronic histiocytic intervillositis. Hematoxylin & eosin, ×10. (**B**). Chronic histiocytic intervillositis showing inflammatory cells (arrows) in the intervillous space. The majority of these cells are histiocytes, with lesser numbers of neutrophils. The chorionic villi are surrounded by fibrin and exhibit necrosis of the trophoblast. These findings have been characteristically present in placentas infected with SARS-CoV-2 [26,27,28]. Hematoxylin & eosin, ×20.

**Figure 2:**
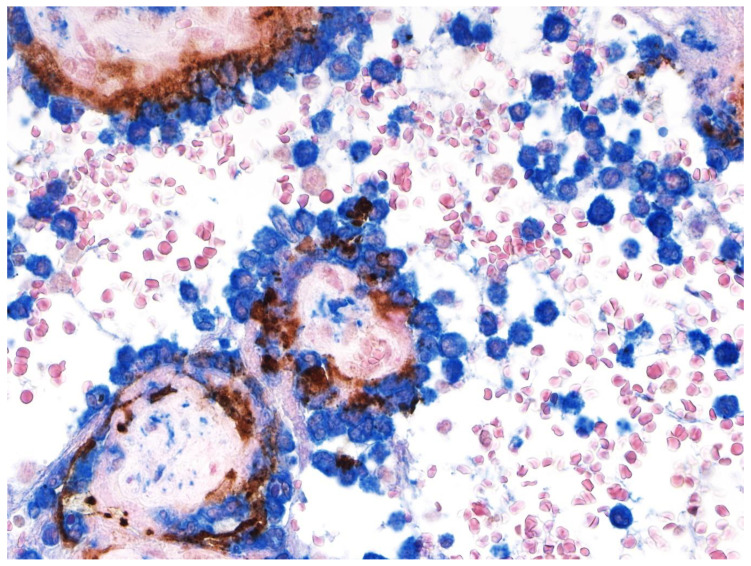
Chronic histiocytic intervillositis. Double-staining immunohistochemistry reveals round-shaped histiocytes in the intervillous space that are strongly positive for CD163 (blue) and surround chorionic villi. The trophoblast layer stains positive (brown) for SARS-CoV-2 nucleocapsid protein. Double staining with antibodies to CD163 and SARS-CoV-2 nucleocapsid protein, nuclear red counterstain. ×40.

Microscopic examination of the placenta using routine hematoxylin & eosin staining revealed the presence of chronic histiocytic intervillositis (Figure 1 and Figure 2), in which the intervillous inflammatory infiltrate was mostly composed of histiocytes admixed with lesser numbers of neutrophils, occurring together with syncytiotrophoblast necrosis and abnormally increased fibrin deposition, all of which involved approximately 30% of the placental parenchyma. The mononuclear cells that composed the majority of the inflammatory infiltrate in the intervillous space stained positive with antibody to CD163, confirming their identity as histiocytes (Figure 2).

**Figure 3:**
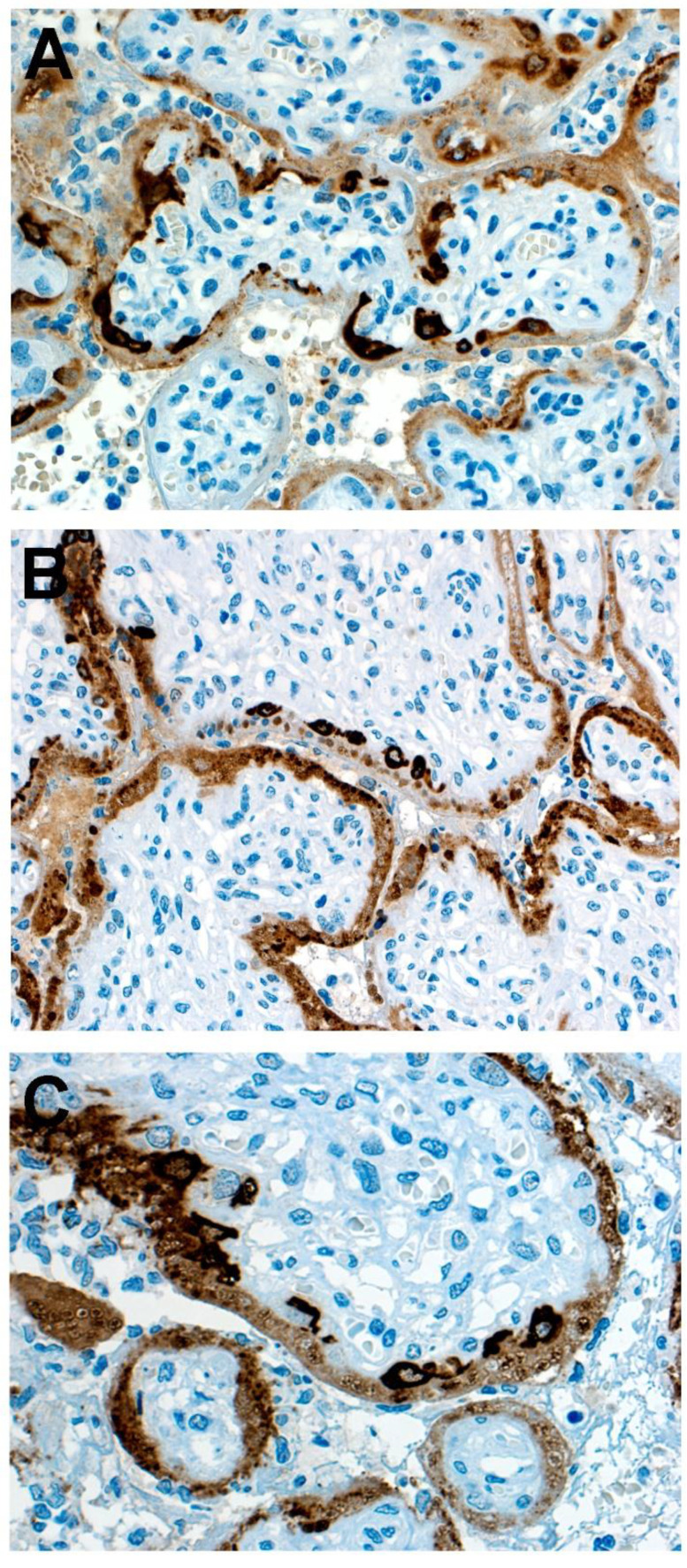
(**A**). Staining with antibody to SARS-CoV-2 nucleocapsid protein is positive (brown) in the trophoblast layer of chorionic villi. The outermost syncytiotrophoblast demonstrates diffuse light brown staining in most villi, and beneath it, dark brown staining of large individual cytotrophoblast cells is present. Note the greater intensity of positive staining in the cytotrophoblast compared with the syncytiotrophoblast. (**B**,**C**). These images demonstrate positive staining cytotrophoblast cells aligned linearly immediately beneath the syncytiotrophoblast, creating a “string of pearls” appearance. As can be seen in all 3 images, some chorionic villi exhibit multiple cytotrophoblast cells that are positive for viral nucleocapsid protein, while other villi have positive staining of only the syncytiotrophoblast. Antibody to SARS-CoV-2 nucleocapsid protein, ×20.

**Figure 4:**
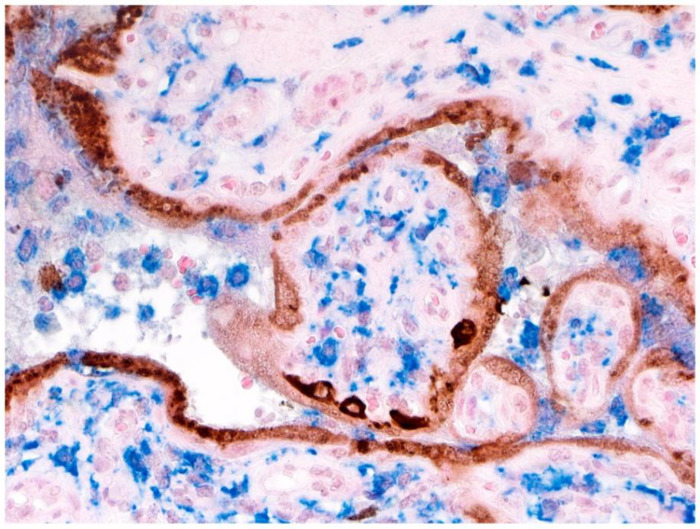
Double-staining using CD163 antibody to macrophages (blue) and antibody to SARS-CoV-2 nucleocapsid protein (brown) demonstrates dark brown-staining cytotrophoblast cells subjacent to light-brown staining syncytiotrophoblast, and their relationship with the villous stromal macrophages (blue), termed Hofbauer cells. Double staining with antibodies to CD163 and SARS-CoV-2 nucleocapsid protein, nuclear red counterstain, ×40.

Similar to other reported placentas infected with SARS-CoV-2 having chronic histiocytic intervillositis and trophoblast necrosis [26,27,28], the syncytiotrophoblast stained diffusely positive for SARS-CoV-2 using immunohistochemistry for viral antigen and RNAscope for viral mRNA. However, unlike previously reported placentas, some cytotrophoblast cells also stained positive for SARS-CoV-2 (Figure 3). The intensity of staining of cytotrophoblast was, in all positive cells, of greater intensity than that of the syncytiotrophoblast. In some villi, cytotrophoblast cells that stained positively with antibody to SARS-CoV-2 nucleocapsid protein were aligned linearly just beneath the syncytiotrophoblast, creating the appearance of a “string of pearls” (Figure 3B,C). Double-staining using antibodies to CD163 and SARS-CoV-2 nucleocapsid protein staining revealed chorionic villous macrophages (Hofbauer cells) in the stroma beneath the cytotrophoblast cells (Figure 4); the cytotrophoblast stained intensely positive for the virus but was negative for CD163, while the Hofbauer cells stained positive for CD163 but were negative for viral staining.

**Figure 5:**
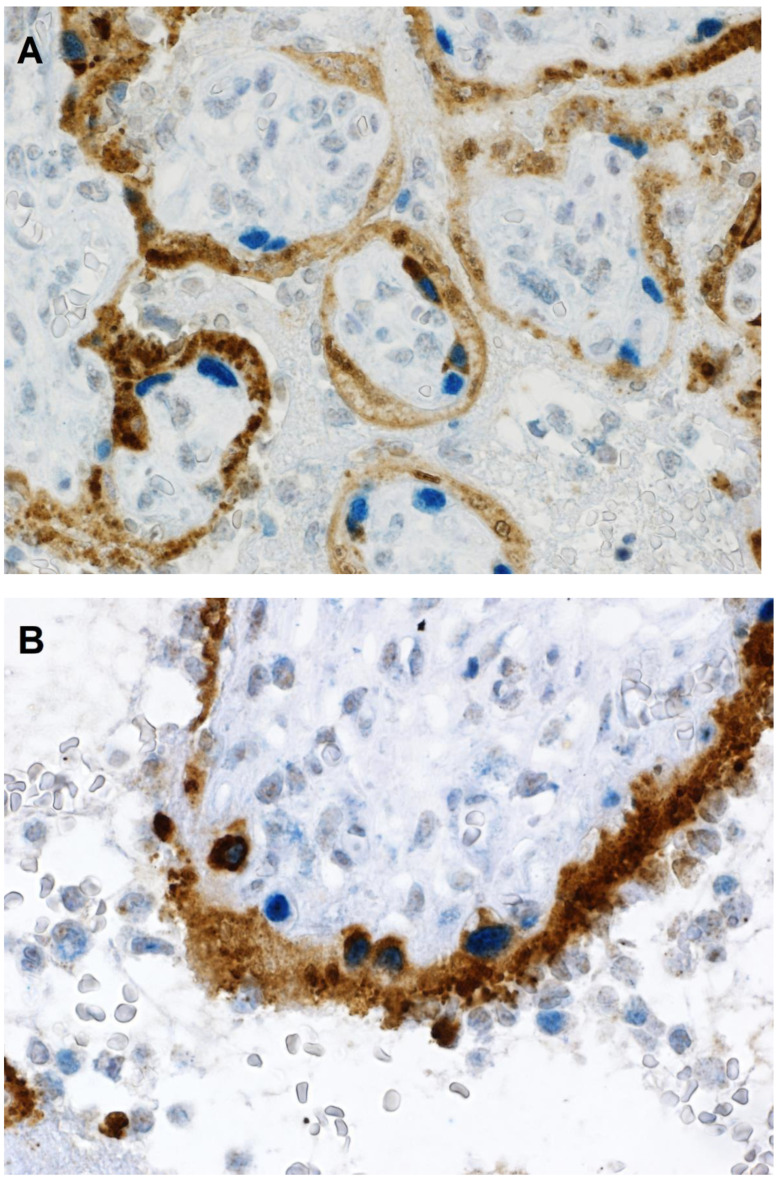
(**A**,**B**). Double-staining immunohistochemistry using GATA3 (blue) and antibody to SARS-CoV-2 nucleocapsid protein (brown). The overlying syncytiotrophoblast are diffusely positive (brown intracytoplasmic staining) for SARS-CoV-2 antigen. In addition, there are cytotrophoblast cells located beneath the syncytiotrophoblast layer that have positive (blue) intranuclear staining for GATA3, and also have intracytoplasmic positivity (brown) for coronavirus. In both images there are cytotrophoblast (blue nuclei) that do not demonstrate intracytoplasmic staining for SARS-CoV-2 antigen. Double staining with antibodies to GATA3 and SARS-CoV-2 nucleocapsid protein, ×40.

**Figure 6:**
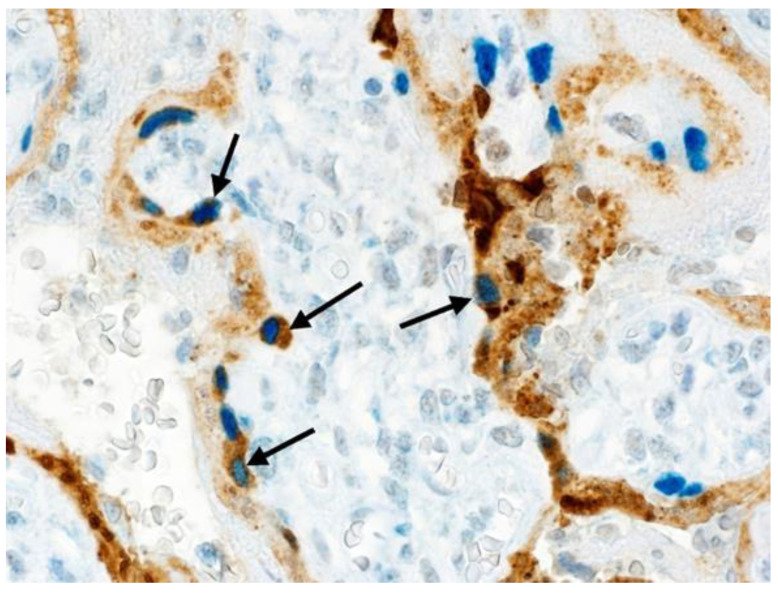
Multiple scattered cytotrophoblast cells (arrows) exhibit intracytoplasmic positivity for SARS-CoV-2 nucleocapsid protein (brown). Cytotrophoblast staining for SARS-CoV-2 nucleocapsid protein is identified using double-staining immunohistochemistry with antibodies to SARS-CoV-2 nucleocapsid protein (brown, cytoplasm) and GATA3 (blue, nucleus). The cytotrophoblast staining positive for viral antigen are in intimate contact with the overlying syncytiotrophoblast, which also stains positive. Note that there are cytotrophoblast cells (blue nuclei, clear cytoplasm) that do not demonstrate intracytoplasmic staining for SARS-CoV-2 antigen. Double staining with antibodies to GATA3 and SARS-CoV-2 nucleocapsid protein, ×40.

**Figure 7:**
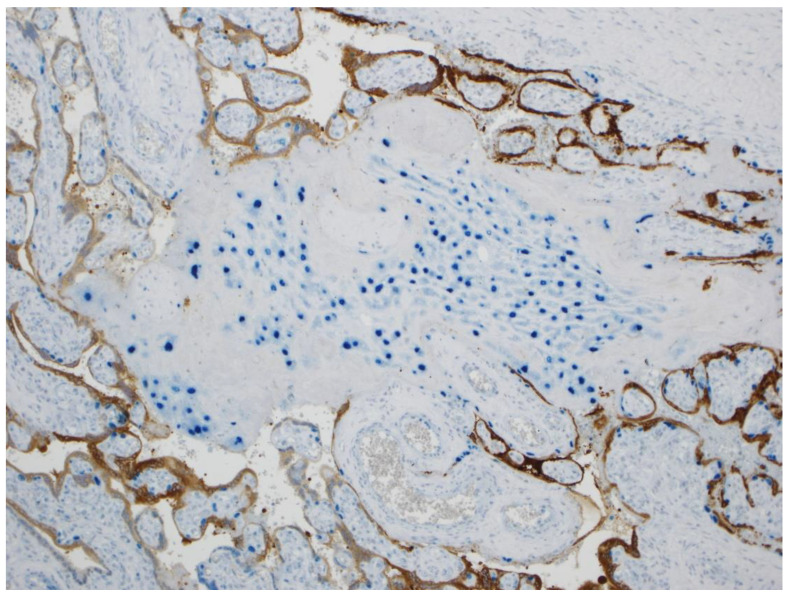
In this double-stained preparation the nuclei of the intermediate trophoblast stain positively (blue) using antibody to GATA3, but the cells are negative for viral staining using antibody to SARS-CoV-2 nucleocapsid protein. Syncytiotrophoblast in the surrounding chorionic villi stain positive (brown) for viral antigen. Double staining with antibodies to GATA3 and SARS-CoV-2 nucleocapsid protein, ×10.

Double-staining immunohistochemistry using antibodies to GATA3 and SARS-CoV-2 nucleocapsid protein revealed that those large cells having the morphological and positional features of cytotrophoblast showed positive (blue) intranuclear staining using GATA3, confirming their identity as cytotrophoblast (Figure 5 and Figure 6). Although the overlying syncytiotrophoblast stained diffusely positive for SARS-CoV-2, not all the underlying cytotrophoblast cells that demonstrated positive intranuclear staining for GATA3 also stained positive for SARS-CoV-2. This suggested that despite their intimate contact with overlying syncytiotrophoblast cells that were positive for SARS-CoV-2 nucleocapsid antigen, many cytotrophoblast cells had not yet been exposed to the viral protein (Figure 5 and Figure 6). In addition to the cytotrophoblast, intermediate trophoblast also stained positive with antibody to GATA3 but was negative for viral antigen staining (Figure 7).

**Figure 8:**
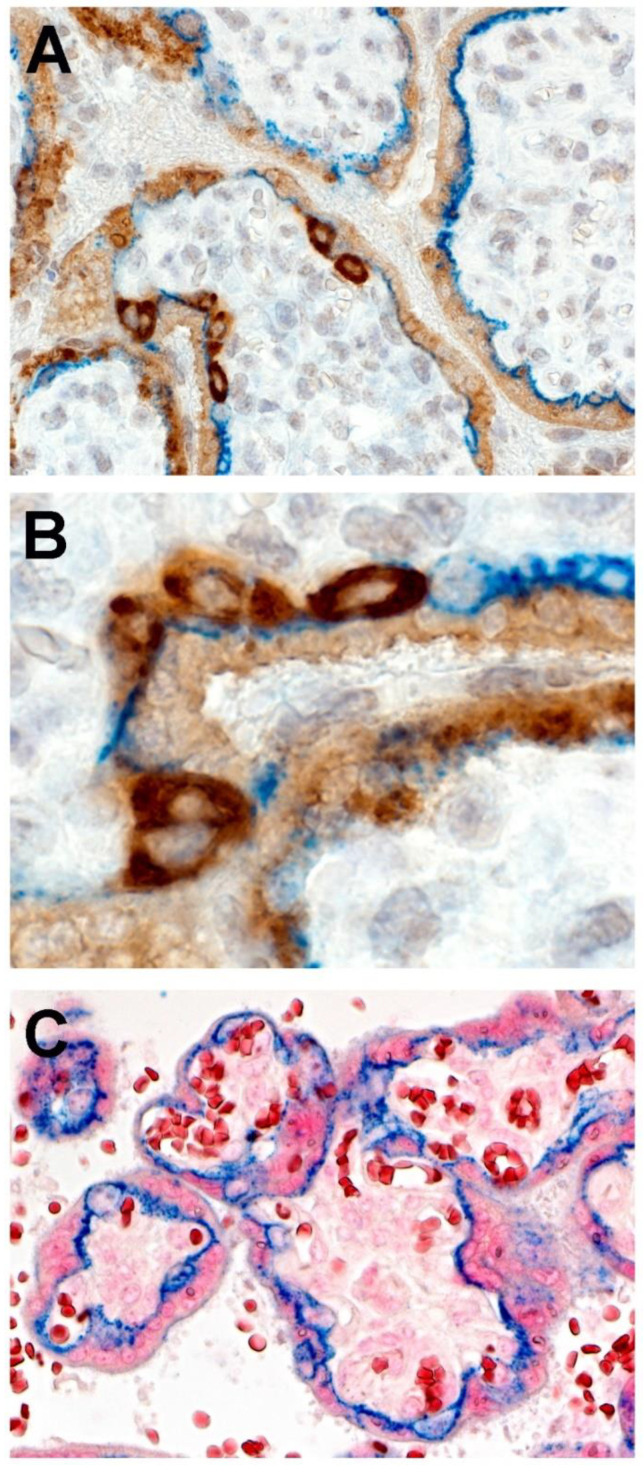
E-cadherin antibody staining of placenta to demonstrate cytotrophoblast. (**A,B**)**.** Double staining with antibodies to E-cadherin and SARS-CoV-2 nucleocapsid protein. Threadlike, delicate and continuous linear staining for E-cadherin (blue) assists in delineating the cytotrophoblast cells staining intensely positive for SARS-CoV-2 nucleocapsid protein from the weaker staining syncytiotrophoblast layer. Image (**B**) is a higher magnification of cytotrophoblast cells present in image (**A**,**C**). A control slide of uninfected placenta showing the surface of the syncytiotrophoblast staining negative for E-cadherin. Linear positive staining (blue) is present at the base of the syncytiotrophoblast, which extends around the outer cell membranes of the cytotrophoblast. Double staining with antibodies to E-cadherin and SARS-CoV-2 nucleocapsid protein. (**A**), Double staining with antibodies to E-cadherin and SARS-CoV-2 nucleocapsid protein, ×20; (**B**), Double staining with antibodies to E-cadherin and SARS-CoV-2 nucleocapsid protein ×100; (**C**), Antibody to E-cadherin and nuclear red counterstain, ×20.

The results of double-staining immunohistochemistry using antibodies to E-cadherin and SARS-CoV-2 nucleocapsid protein revealed a linear pattern of delicate strands that stained positive (blue) for E-cadherin within the trophoblast layer (Figure 8A,B). These strands delineated the syncytiotrophoblast and cytotrophoblast layers. Using this method, the positive staining cells of the outermost syncytiotrophoblast and the underlying cytotrophoblast cells were readily discernible (Figure 8A,B), and were similar to control slides in which linear positive staining for E-cadherin was present at the base of the syncytiotrophoblast and extended around the outer cell membranes of the cytotrophoblast (Figure 8C). 

**Figure 9:**
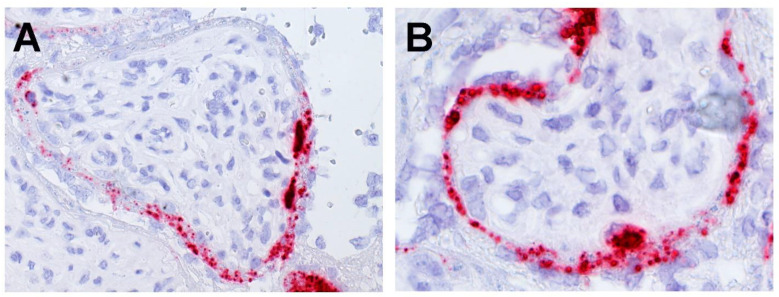
**A,B.** Large cells immediately beneath the syncytiotrophoblast layer that are morphologically consistent with cytotrophoblast show strong positive staining using RNAscope for SARS-CoV-2 spike protein mRNA. The syncytiotrophoblast also stains positive, but not as intensely. (**A**), ×20; (**B**), ×40.

RNAscope staining demonstrated positive staining for SARS-CoV-2 spike protein mRNA in large cells beneath and in contact with the syncytiotrophoblast that were morphologically consistent with cytotrophoblast (Figure 9). Similar to the immunohistochemical staining patterns, RNAscope revealed that the cytotrophoblast stained with much greater intensity than did the syncytiotrophoblast.

## 4. Discussion 

In past epidemics of emerging infections, the pathological analysis of placentas from infected pregnant women has proven to be a highly informative technique for understanding mechanisms of transmission of viral agents to the fetus. The COVID-19 pandemic that was first recognized in Wuhan, China has had a profound effect on pregnant women and birthing throughout the globe. In order to ensure the best clinical practice, institute effective infection control, and advance scientific knowledge and possible methods for diagnosis and treatment, it was important to determine whether the SARS-CoV-2 virus infecting pregnant women could result in vertically acquired infections, and if it did, how and when transmission was occurring. In the early phases of the pandemic, pathology investigations of placentas from pregnant women with SARS-CoV-2 infection were initiated to help understand the effects of the virus on the placenta and the potential mechanisms of maternal-fetal transmission. However, multiple conflicting results were found in these early studies that proved to be confusing, as a variable and inconsistent spectrum of findings were described. These included fetal and maternal vascular malperfusion, thromboses, hemorrhage, increased fibrin, a variety of inflammatory abnormalities and combinations of these processes [31,32,33]. The absence of specific findings related to COVID-19 in placentas from infected women was suggested in several articles [34,35,36], including one entitled “SARS-CoV-2 can infect the placenta and is not associated with specific placental histopathology…” [35], adding to this uncertainty. However, because the large majority placentas that were examined in these studies were not infected with SARS-CoV-2, and the large majority of neonates tested negative for coronavirus infection, the pathology findings were largely derived from examining uninfected placentas from uninfected fetuses and neonates. By taking a different approach to analyzing the effects of SARS-CoV-2 on the placenta, Schwartz and colleagues [26,27] used coronavirus infection of the mother and fetus as inclusion criteria and found that the placentas from 11 infected maternal-fetal dyads demonstrated a consistent and unique pattern of three highly unusual pathology abnormalities that were occurring concurrently, and which were associated with intrauterine transplacental infection. The placentas from six live-born infected neonates and five stillborn infants contained the same group of pathology findings: chronic histiocytic intervillositis, trophoblast necrosis and positive staining of the syncytiotrophoblast for SARS-CoV-2 using immunohistochemistry and/or RNA in situ hybridization. In addition to all 11 placentas having these concurrent abnormalities, most placentas also showed increased fibrin deposition, including some with massive perivillous fibrin deposition [27]. The simultaneous occurrence of these abnormalities was beyond coincidence and has subsequently been reported from additional infected placentas [37,38,39,40,41,42].

The syncytiotrophoblast constitutes the most important cell type in the protective maternal-fetal interface, serving a critical role in protecting the fetus from infectious agents. Because it is in direct physical contact with maternal blood in the intervillous space, the syncytiotrophoblast is potentially vulnerable to all pathogens circulating in the mother’s circulation [27]. Based upon the observations made in recent articles describing the pathology findings from infected placentas, it can be confidently stated that the syncytiotrophoblast is the most frequent cell type composing the maternal-fetal interface to be infected with SARS-CoV-2 [28]. The syncytiotrophoblast expresses the major cell surface receptor for SARS-CoV-2, angiotensin-converting enzyme 2 (ACE2), throughout gestation [43,44]. Because of its strategic location on the surface of the syncytiotrophoblast, this receptor is exposed directly to maternal blood that circulates through the intervillous space, and which may contain SARS-CoV-2 in infected pregnant women with viremia. In addition to ACE2, the syncytiotrophoblast also expresses the SARS-CoV-2 entry factors transmembrane protease serine 2 (TMPRSS2), and furin [44,45]. Recent evidence has suggested that in contrast to 1st and 2nd trimester placentas, there is low expression of the ACE2 viral receptor on placental trophoblast cells during the later stages of pregnancy [46,47], potentially accounting for the relatively low rate of transplacental transmission that occurs with SARS-CoV-2. However, there are data suggesting that term placentas from pregnant women infected with SARS-CoV-2 have increased levels of the ACE2 protein [48]. In addition to the syncytiotrophoblast, small numbers of infected placentas have been found to demonstrate positive immunohistochemical staining for SARS-CoV-2 in other fetal-derived placental cells including Hofbauer cells and villous capillary endothelial cells [29].

This report demonstrates that the cytotrophoblast can be involved in placental SARS-CoV-2 infection, adding a new cell type to those fetal-derived cells of the chorionic villus that stain positively for the virus. In addition to identifying cytotrophoblast staining for SARS-CoV-2 using immunohistochemistry and RNA in situ hybridization methods, we used antibodies to GATA3 [49] and E-cadherin [50,51] to distinguish cytotrophoblast from syncytiotrophoblast cells, and provide the first detailed description of the detection of SARS-CoV-2 in the cytotrophoblast. In this placenta, the syncytiotrophoblast was also positive using both immunohistochemical and RNA in situ hybridization methods, but Hofbauer cell and villous capillary endothelial staining was not present. This observation is interesting in view of the recent demonstration by Schwartz et al. [29] that among 22 placentas infected with SARS-CoV-2, four (18%) placentas had positive staining of Hofbauer cells and two (9%) placentas demonstrated staining of villous capillary endothelial cells for the virus. Because the syncytiotrophoblast and cytotrophoblast are in intimate physical contact with one another, and the villous cytotrophoblast has been demonstrated to stain using antibody to the ACE2 receptor protein [52,53], it appears likely that our observation of the simultaneous presence of SARS-CoV-2 antigen and nucleic acid in both cell types indicates cell-to-cell passage of the virus. The trophoblast basement zone that separates these cell types from the underlying villous stroma and its constituents (Hofbauer cells and capillaries) may serve as a protective barrier the inhibits SARS-CoV-2 from entering the villous core. However, there are no data currently available on the dynamics of viral spread within the chorionic villus.

The staining intensity of the cytotrophoblast cells using RNAscope as well as antibody to the SARS-CoV-2 nucleocapsid protein was much greater than that of the overlying syncytiotrophoblast, and although it cannot be confirmed from this study, may represent higher levels of the viral mRNA and antigen within these cells. This observation is interesting following the recent report from Lu-Culligan et al. [48] that cytotrophoblasts, in addition to trophoblast stem cells, rather than syncytiotrophoblast or Hofbauer cells, were the most vulnerable cells to direct SARS-CoV-2 infection in vitro using immortalized cell lines and primary isolated placental cells. The staining of cytotrophoblast in this placenta for SARS-CoV-2 was intermittent, with some cells staining positive and the adjacent cells remaining unstained. However, immunohistochemistry revealed that in some chorionic villi, a formation of adjacent cytotrophoblast cells in linear arrays stained positively for SARS-CoV-2 nucleocapsid protein—we termed this appearance a “string of pearls”. Typically, when a chorionic villus exhibited cytotrophoblast that stained positive for SARS-CoV-2 nucleocapsid antibody, there were multiple cells that stained. In contrast, there were chorionic villi that exhibited diffuse staining of the syncytiotrophoblast for SARS-CoV-2 nucleocapsid protein in which there was no staining of the cytotrophoblast. Based upon our findings, we believe that the syncytiotrophoblast is exposed to and becomes positive for SARS-CoV-2 prior to involvement of the cytotrophoblast. 

In their role as progenitor cells, two or more villous cytotrophoblast cells undergo fusion to form the terminally differentiated syncytiotrophoblast. Cytotrophoblast divide constantly throughout pregnancy and are in one of the phases of the mitotic cell cycle–G_0_, G_1_, S, G_2_ or M [54]. The cytotrophoblast rests directly on a basal lamina, the other side of which is in contact with the mesenchymal stromal cells, Hofbauer cells and fetal blood vessels that make up the villous core. Cytotrophoblast cells have fine processes that are contiguous with surrounding cells, and as gestation progresses, this layer of cells becomes thinner but still maintains most of its structural integrity [55]. The observation in this placenta that the cytotrophoblast demonstrated SARS-CoV-2 nucleocapsid protein and mRNA is interesting and raises several important questions. How frequently is the cytotrophoblast involved with SARS-CoV-2 in infected placentas? As the syncytiotrophoblast is in direct contact with maternal blood at its apical aspect and with cytotrophoblast on its basal border, what is the mechanism for the cytotrophoblast to acquire viral material? Why does the cytotrophoblast stain more intensely than does the syncytiotrophoblast? Is the intense positive staining of cytotrophoblast for SARS-CoV-2 nucleocapsid protein and mRNA that was observed indicative of the virus replicating in the cytotrophoblast? And could the close and intimate contact of the cytotrophoblast with the core of the chorionic villus expedite transmission of the virus across the maternal-placental barrier? When cytotrophoblast cells that stain positive for viral protein and mRNA divide and eventually fuse to form the syncytiotrophoblast, what happens to the viral material? These and other interesting questions must await further research. 

## Data Availability

Data sharing is not applicable to this article.
